# The prognostic impact of squamous differentiation in bladder cancer after radical cystectomy: a propensity score-matched comparative analysis of pure urothelial carcinoma, urothelial carcinoma with squamous differentiation, and squamous cell carcinoma

**DOI:** 10.3389/fonc.2026.1890852

**Published:** 2026-07-07

**Authors:** Miaolin Guo, Yu Zhou, Xinjie Lin, Zhihong Xu, Yuxuan You

**Affiliations:** 1 Hainan Academy of Medical Sciences, Hainan Medical University, Haikou, China; 2 Department of Urology, Zhangzhou Affiliated Hospital of Fujian Medical University, Zhangzhou, Fujian, China; 3 Department of Anesthesiology, The 909th Hospital, School of Medicine, Xiamen University, Zhangzhou, Fujian, China; 4 Department of Critical Care Medicine, Mindong Hospital Affiliated to Fujian Medical University, Fuan, Fujian, China; 5 922th Hospital of Joint Logistics Support Force, PLA, Hengyang, Hunan, China

**Keywords:** bladder cancer, prognosis, propensity score matching, radical cystectomy, squamous differentiation

## Abstract

**Objectives:**

To evaluate the prognostic impact of squamous differentiation in bladder cancer by comparing clinicopathological characteristics and survival outcomes among patients with pure urothelial carcinoma (UC), urothelial carcinoma with squamous differentiation (UCSD), and pure squamous cell carcinoma (SCC) treated with radical cystectomy (RC).

**Methods:**

A single-center, retrospective cohort analysis was conducted. Patients who underwent RC for bladder cancer between January 2015 and June 2024 were identified and stratified into three groups based on final pathology: pure UC, UCSD, and SCC. Clinicopathological features were compared, and overall survival (OS) was analyzed using Kaplan-Meier methods and multivariable Cox regression. To control for baseline imbalances, 1:1 propensity score matching (PSM) was performed, matching for age, sex, surgical approach, muscle invasion status, and lymph node status.

**Results:**

Among 312 eligible patients, there were 236 with pure UC,60 with UCSD, and 16 with SCC.In unadjusted analysis,patients with UCSD and SCC presented with more advanced disease (higher rates of stage III+IV, muscle invasion, and lymph node metastasis;*P* < 0.05) and had significantly worse 5-year OS (42.1% and 38.9%, respectively) compared to the pure UC group (62.5%;log-rank *P* < 0.001). After 1:1 propensity score matching, baseline characteristics were well-balanced across the three groups. Pairwise log-rank comparisons demonstrated that overall survival was significantly worse in both the UCSD and SCC groups compared to the pure UC group (UC vs.UCSD: HR = 1.52,95% CI:1.18–1.96,*P* = 0.002; UC vs.SCC: HR = 1.78,95% CI:1.25–2.54,*P* = 0.001).However, no significant difference was observed between the UCSD and SCC groups (HR = 1.17,95% CI:0.79–1.73,*P* = 0.622).In the matched cohort,the 5-year OS rates were 59.5% for pure UC, 45.8% for UCSD, and 42.6% for SCC.In multivariable Cox regression (full cohort), older age(HR = 1.03,95% CI:1.02–1.04,*P* < 0.001), muscle invasion (HR = 2.25,95% CI: 1.75–2.89,*P* < 0.001), positive lymph node status (HR = 2.18,95% CI: 1.65–2.88,*P* < 0.001), and pathological subtype were independent predictors of worse OS. Specifically, compared to pure UC, UCSD (HR = 1.52, 95% CI: 1.18–1.96,*P* = 0.002) and SCC (HR = 1.78,95% CI: 1.25–2.54,*P* = 0.001) were independently associated with worse OS.

**Conclusions:**

Patients with UCSD and SCC present with more advanced disease at diagnosis, leading to poorer unadjusted survival. After rigorous adjustment for key clinicopathological confounders, squamous differentiation remained an independent risk factor for worse overall survival following radical cystectomy, with both UCSD and pure SCC associated with significantly poorer prognosis compared to pure UC, whereas no significant difference existed between UCSD and SCC. These findings indicate that squamous differentiation confers an intrinsically more aggressive tumor biology that is not fully explained by stage or nodal status alone.

## 1 Propensity score matching;prognosis

Bladder cancer ranks among the most prevalent malignancies of the urinary tract worldwide, contributing significantly to global cancer incidence and mortality and posing a substantial public health burden (1, 2). Urothelial carcinoma(UC) constitutes the predominant histological subtype, accounting for over 90% of all bladder cancer cases (3). The remaining spectrum encompasses non-urothelial histologies and/or divergent differentiation, with pure squamous cell carcinoma (SCC) representing 2.4%–6.7% of cases (4, 5); this proportion rises markedly in certain geographical regions, such as areas endemic for schistosomiasis. Furthermore, approximately 30% of UCs exhibit histological variants, among which squamous differentiation (SqD) is one of the most frequently observed patterns (6).

Accumulating evidence suggests that bladder cancers harboring squamous elements demonstrate more aggressive clinical behavior compared to pure conventional UCs. Multiple retrospective studies indicate that patients with urothelial carcinoma with squamous differentiation (UCSD) or pure SCC often present at diagnosis with more advanced pathological stage, higher rates of muscle invasion, and increased lymph node metastasis, culminating in significantly worse overall survival (OS) relative to their conventional UC counterparts (7, 8). For instance, UCSD has been identified as an independent predictor of disease progression and cancer-specific mortality (9).

Nevertheless, considerable controversy persists regarding whether squamous differentiation per se serves as an independent prognostic determinant beyond established factors such as TNM stage and nodal status. Some investigators posit that the observed inferior outcomes may largely be attributable to more advanced disease at presentation rather than an intrinsically aggressive biology inherent to the histological variant itself (10). Elucidating the true prognostic impact of squamous differentiation is therefore critical for accurate risk stratification, informing therapeutic intensity—including decisions regarding radical cystectomy (RC) and adjuvant therapy—and optimizing surveillance protocols for affected patients.

Current clinical decision-making in bladder cancer predominantly relies on traditional TNM staging and WHO grading systems, which have yet to adequately incorporate information on histological variants. Although numerous studies have addressed this issue, many are limited by small sample sizes—particularly for the rarer SCC subtype—single-center retrospective designs, and a lack of rigorous methodology to control for confounding factors. Few investigations have systematically employed methods such as propensity score matching (PSM) to mitigate baseline imbalances, potentially overestimating the independent effect of histology.

To address these gaps, this large single-center retrospective study aimed to: 1) compare clinicopathological characteristics among three histological groups—pure UC, UCSD, and pure SCC; 2) analyze differences in OS across these subtypes; and 3) specifically investigate, using multivariable Cox regression and PSM to adjust for key confounders, whether squamous differentiation constitutes an independent predictor of OS following RC. The findings are expected to provide more robust evidence to guide individualized risk assessment and management strategies for bladder cancer patients with squamous differentiation.

## 2 Materials and methods

### 2.1 Study design and patient cohort

This was a single−center, retrospective cohort study conducted in accordance with the Declaration of Helsinki. The study protocol was reviewed and approved by the Institutional Review Board of Fujian Medical University Affiliated Hospital. The requirement for written informed consent was waived due to the retrospective nature of the study. All patient data were anonymized and de−identified prior to analysis. We systematically reviewed the medical records of all consecutive patients who underwent radical cystectomy (RC) with bilateral pelvic lymph node dissection for bladder cancer in the Department of Urology at Fujian Medical University Affiliated Hospital between January 1, 2015, and June 30, 2024.

### 2.2 Patient selection criteria

The inclusion criteria were as follows: (1) age >18 years at the time of surgery; (2) pathological confirmation of UC, UCSD, or pure SCC of the bladder in the RC specimen; (3) no evidence of distant metastasis (M0) based on preoperative imaging (chest and abdominal computed tomography or magnetic resonance imaging) and intraoperative findings; (4) availability of complete clinicopathological data and follow−up information.

Patients were excluded for any of the following reasons: (1) presence of distant metastasis (M1 stage); (2) pathological diagnosis of variant histologies other than squamous differentiation (e.g., glandular differentiation, micropapillary, plasmacytoid, sarcomatoid, small−cell/neuroendocrine carcinoma) or non−urothelial malignancies (e.g., pure adenocarcinoma); (3) history of any prior malignancy within the past 5 years (except for non−melanoma skin cancer); (4) receipt of palliative RC or perioperative mortality (death within 30 days post−surgery).

### 2.3 Data collection and definitions

Data were extracted retrospectively from electronic medical records using a standardized, pre-piloted data collection form. The collected variables included:

Demographic and clinical characteristics: age at surgery, sex, body mass index (BMI), smoking history (categorized as never-smoker or smoker with a cumulative history of >10 pack-years), and preoperative American Society of Anesthesiologists (ASA) physical status score.

Preoperative laboratory parameters: the most recent preoperative hemoglobin level (anemia was defined as hemoglobin <120 g/L) and serum creatinine value. The estimated glomerular filtration rate (eGFR) was calculated using the Chronic Kidney Disease Epidemiology Collaboration (CKD-EPI) equation, with renal insufficiency defined as eGFR <60 mL/min/1.73 m².

Surgical and perioperative data: year of surgery, surgical approach (open, laparoscopic, or robot-assisted), operative time, estimated blood loss, type of urinary diversion (ileal conduit vs. orthotopic neobladder), and postoperative complications graded according to the Clavien-Dindo classification within 90 days.

Pathological Review and Methodological Rigor: To ensure the highest level of diagnostic accuracy and data reliability, a centralized pathological review process was implemented. All histological slides from radical cystectomy (RC) specimens were independently re-reviewed by two dedicated genitourinary pathologists who were blinded to the original diagnosis and clinical outcomes. The presence of squamous differentiation was defined based on definitive morphological features, including keratin pearl formation and/or intercellular bridges. Cases with discrepancies were resolved through joint review and consensus discussion in a multidisciplinary tumor board.

Inter-observer agreement was formally assessed using Cohen’s kappa coefficient, yielding a value of *κ* = 0.87 (95% CI: 0.82–0.92), which indicates excellent agreement beyond chance.

Based on this review, patients were stratified into three groups:

Pure UC: tumors composed exclusively of conventional urothelial carcinoma without any squamous component.
UCSD: tumors exhibiting definitive foci of squamous differentiation within an otherwise urothelial carcinoma, irrespective of the quantitative proportion.
Pure SCC: tumors composed entirely of squamous cell carcinoma with no urothelial component.

Additional pathological features recorded included: pathological stage according to the American Joint Committee on Cancer (AJCC) 8th edition TNM staging system (11); tumor grade based on the 2016 World Health Organization (WHO) classification; presence of lymphovascular invasion (LVI) (12); surgical margin status (positive defined as carcinoma *in situ* or invasive cancer at the resection margin); multifocality; and maximum tumor diameter. Muscle-invasive disease was defined as pathological T stage ≥T2.

Adjuvant therapy: administration of postoperative adjuvant chemotherapy (yes/no) and the specific regimen used were recorded.

### 2.4 Follow-up and outcome definitions

All patients were followed according to a standardized institutional protocol. Follow-up visits were scheduled at 3-month intervals for the first 2 years, every 6 months for the next 3 years, and annually thereafter. Each visit included history, physical examination, serum chemistry, urinary cytology, and cross-sectional imaging of the chest/abdomen/pelvis. Cystoscopy was performed annually in patients with orthotopic neobladders.

The primary endpoint was overall survival (OS), defined as the time from the date of RC to death from any cause. Living patients were censored at the date of the last follow-up (June 30, 2025). Secondary endpoints included:Cancer-specific survival (CSS): time from RC to death attributable to bladder cancer or its direct complications.Recurrence-free survival (RFS): time from RC to the first evidence of local recurrence (in the tumor bed, regional lymph nodes, or urinary diversion) or distant metastasis, confirmed by imaging or biopsy.Patients who died from causes unrelated to bladder cancer without prior recurrence were censored for RFS analysis at the date of death.

### 2.5 External validation

To assess generalizability, we performed a brief external validation using the Surveillance, Epidemiology, and End Results (SEER) database (2004–2020). Patients with pure UC, UCSD, and pure SCC who underwent radical cystectomy were identified using ICD-O-3 histology codes (8120/3 for UC, 8070/3 for SCC; UCSD was identified by histology code 8120/3 with squamous differentiation noted in the text-derived variable). The same inclusion/exclusion criteria and propensity score matching (age, sex, T stage, nodal status) were applied. Survival analyses were repeated, and results were compared to the primary cohort.

### 2.6 Statistical analysis

Statistical analyses were performed using SPSS version 26.0 (IBM Corp.) and R software (version 4.2.2, The R Foundation). All tests were two-sided, and a *P* < 0.05 was considered statistically significant.

Descriptive Statistics: Continuous variables with normal distribution are presented as mean ± standard deviation and were compared using one-way analysis of variance (ANOVA). Non-normally distributed data are presented as median (interquartile range, IQR) and were compared using the Kruskal-Wallis H test. Categorical variables are presented as frequency (percentage) and were compared using the Chi-square test or Fisher’s exact test, as appropriate.

Survival Analysis: Kaplan-Meier survival curves were generated for OS, CSS, and RFS, and differences among the three pathological groups were compared using the log-rank test.

Propensity Score Matching (PSM): To minimize confounding bias due to baseline imbalances, we performed pairwise 1:1 nearest-neighbor propensity score matching without replacement for each pair of histological groups: pure UC vs. UCSD, pure UC vs. SCC, and UCSD vs. SCC, using the “MatchIt” package in R. The pathological subtype was the grouping variable. Matching covariates included age, sex, surgical approach, pathological T stage (dichotomized as ≥T2 vs. <T2), lymph node status (positive vs. negative), and maximum tumor diameter (continuous). A caliper width of 0.02 of the standard deviation of the logit of the propensity score was applied. Standardized mean differences (SMD) were calculated for all covariates before and after matching, with an SMD <0.10 indicating good balance. Survival analyses were repeated in the matched cohort.

Due to the relatively small number of pure SCC cases (n=16) in our institutional cohort, all analyses involving comparisons with the SCC group were considered exploratory. To improve the robustness of conclusions regarding pure SCC, we performed external validation using the SEER database, which included a substantially larger SCC cohort (n=227). Findings specific to pure SCC are interpreted with appropriate caution throughout this manuscript.

Multivariable Cox Regression Analysis: To identify independent predictors of survival, multivariable Cox proportional hazards models were constructed for the entire cohort and the PSM-matched cohort separately. Variables entered into the initial model included pathological subtype (with pure UC as reference), age (continuous), sex, surgical approach, muscle invasion status, lymph node status, LVI status, and adjuvant chemotherapy. Model assumptions were checked, and multicollinearity was assessed using variance inflation factors (all VIFs < 2.0). Hazard ratios (HR) with 95% confidence intervals (CI) were calculated.

Handling of Missing Data: The proportion of missing data for all variables was <2%. Missing data were handled using multiple imputation by chained equations (MICE) for the primary regression analyses. A complete-case analysis was also performed as a sensitivity check, and results were consistent.

Subgroup analysis of muscle-invasive bladder cancer (MIBC): Given the significant imbalance in the proportion of muscle-invasive disease across histologic groups, a subgroup analysis was performed exclusively in patients with pathological stage≥T2(MIBC). In this subgroup, we compared the distribution of advanced T stage(T3/T4vs.T2) among the three histologic groups using the Chi-square test. Additionally, the prognostic impact of histologic subtype on overall survival (OS) was reassessed within the MIBC subgroup using multivariable Cox regression models, adjusting for T stage (categorized as T2vs.T3/T4), lymph node status, and other predefined confounders.

## 3 Results

### 3.1 Patient selection process and baseline characteristics

**Figure 1:**
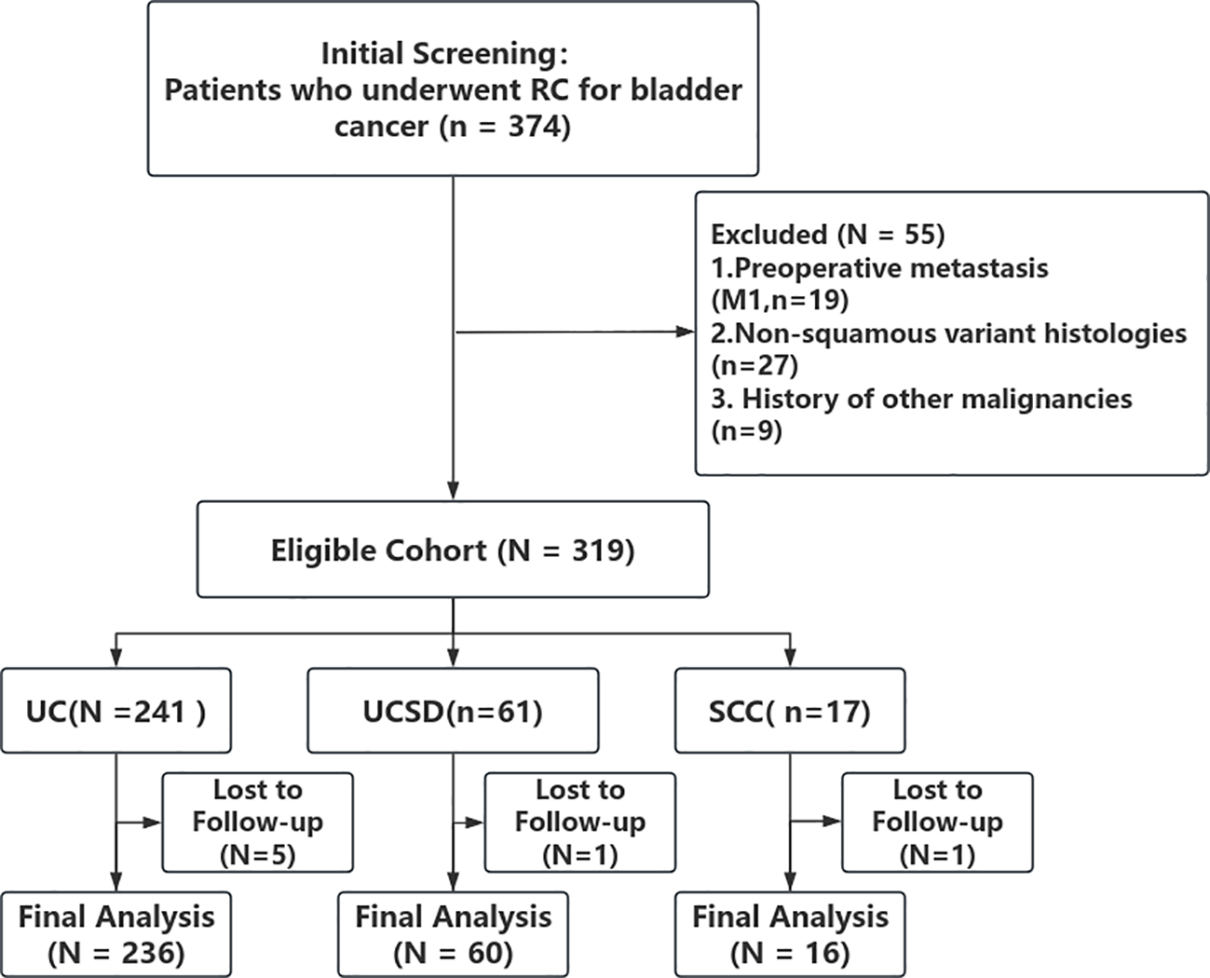
STROBE flowchart of patient selection.

A total of 374 consecutive patients underwent RC for bladder cancer at our institution between January 2015 and June 2024. After applying the inclusion and exclusion criteria, 55 patients were excluded (19 due to preoperative metastasis [M1], 27 due to non-squamous variant histology, and 9 due to concurrent other malignancies). Of the remaining 319 patients, 7 (2.2%) were lost to follow-up. Consequently, 312 patients with complete follow-up data constituted the final analytic cohort (**Figure 1**). Based on central pathological review, patients were stratified into three groups: pure UC (n=236), UCSD (n=60), and pure SCC (n=16).

The baseline clinicopathological characteristics of the three patient groups are compared in **Table 1**. Regarding demographic features, the SCC group had the highest median age and a significantly greater proportion of female patients compared to the other two groups (*P* = 0.012).In terms of key pathological features reflecting tumor aggressiveness, the two groups with squamous differentiation (UCSD and SCC) demonstrated significantly higher malignant potential. Compared to the pure UC group, both the UCSD and SCC groups had higher proportions of advanced stage (Stage III+IV) disease (*P* = 0.002), higher muscle invasion rates (*P* < 0.001), and higher lymph node metastasis rates (*P* = 0.011). Furthermore, the rate of lymphovascular invasion was significantly higher in the UCSD group than in the other two groups (*P* = 0.012).In relation to tumor burden, both the UCSD and SCC groups presented with larger maximum tumor diameters (*P* < 0.001). However, there were no significant differences among the three groups in systemic status indicators, including the prevalence of preoperative anemia and renal insufficiency. Additionally, no statistically significant differences were observed among the groups in body mass index (*P* = 0.127), smoking history (*P* = 0.773), ASA score (*P* = 0.135), surgical approach (*P* = 0.151), type of urinary diversion (*P* = 0.237), or positive surgical margin rate (*P* = 0.787).

**Table 1:** Comparison of baseline clinicopathological characteristics among the three groups.

Characteristic	PUC (n=236)	UCSD (n=60)	SCC (n=16)	Statistic	*P*
Demographic features					
Age (years), median (IQR)	66 (58-71)	67 (59-74)	69 (61-76)	H=5.19	0.189
Male, n (%)	177 (75.0)	48 (80.0)	10 (62.5)	χ²=8.92	0.012
BMI (kg/m²), median (IQR)	24.5 (22.4-26.7)	24.0 (21.8-26.2)	23.6 (21.3-25.9)	H=4.12	0.127
Smoking History (>10 pack-years), n (%)	104 (44.1)	27 (45.0)	8 (50.0)	χ²=0.52	0.773
ASA Score ≥3, n (%)	63 (26.7)	18 (30.0)	6 (37.5)	χ²=4.01	0.135
Preoperative laboratory parameters					
Anemia (Hb <120 g/L), n (%)	68 (28.8)	19 (31.7)	5 (31.3)	χ²=1.45	0.485
eGFR <60 mL/min/1.73m², n (%)	73 (30.9)	16 (26.7)	5 (31.3)	χ²=1.85	0.396
Surgical features					
Surgical Approach (Minimally Invasive), n (%)	132 (55.9)	31 (51.7)	7 (43.8)	χ²=3.78	0.151
Urinary Diversion (Orthotopic Neobladder), n (%)	106 (44.9)	25 (41.7)	6 (37.5)	χ²=2.88	0.237
Pathological features					
Stage III+IV, n (%)	88 (37.3)	29 (48.3)	8 (50.0)	χ²=12.64	0.002
Muscle Invasion (≥pT2), n (%)	115 (48.7)	49 (81.7)	14 (87.5)	χ²=95.61	<0.001
Lymph Node Positive, n (%)	38 (16.1)	13 (21.7)	5 (31.3)	χ²=9.11	0.011
Lymphovascular Invasion Positive, n (%)	26 (11.0)	11 (18.3)	3 (18.8)	χ²=8.93	0.012
Positive Surgical Margin, n (%)	11 (4.7)	3 (5.0)	1 (6.3)	χ²=0.48	0.787
Maximal Tumor Diameter (cm), median (IQR)	2.7 (1.8-4.0)	3.6 (2.6-5.1)	4.1 (2.9-5.8)	H=68.32	<0.001

PUC, Pure Urothelial Carcinoma; UCSD, Urothelial Carcinoma with Squamous Differentiation; SCC, Squamous Cell Carcinoma; IQR, Interquartile Range; BMI, Body Mass Index; ASA, American Society of Anesthesiologists; Hb, Hemoglobin; eGFR, estimated Glomerular Filtration Rate.

### 3.2 Survival analysis

**Figure 2:**
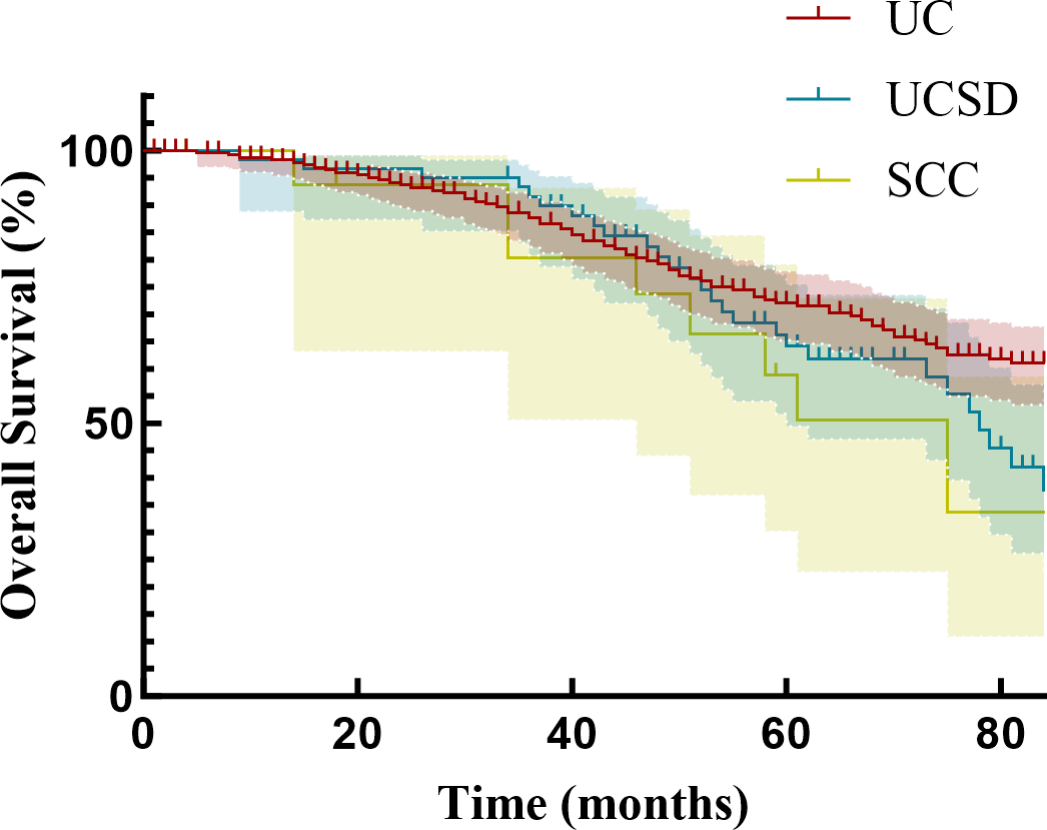
Kaplan-Meier survival curves for overall survival in the overall cohort of bladder cancer patients stratified by pathological type.

All patients were followed up, with a median follow-up time of 51.0 months (6–102 months). In the overall cohort, Kaplan-Meier survival analysis revealed significant differences in overall survival among the three groups (Log-rank *χ²*= 24.43,*P* < 0.001; see **Figure 2**). The 5-year overall survival rates were 42.1% for the UCSD group and 38.9% for the SCC group, both of which were significantly lower than the 62.5% rate observed in the pure UC group. Moreover, a statistically significant difference in survival curves was also present between the UCSD group and the SCC group (Log-rank χ²=9.767,*P* = 0.002).

To control for confounding bias arising from baseline imbalances, we performed 1:1 nearest-neighbor propensity score matching using the MatchIt package. Matching variables included age, sex, surgical approach, muscle invasion status, and lymph node status. After matching, the standardized mean differences for all covariates were below 10% across the three groups, indicating good balance was achieved and the groups were well balanced.

**Figure 3:**
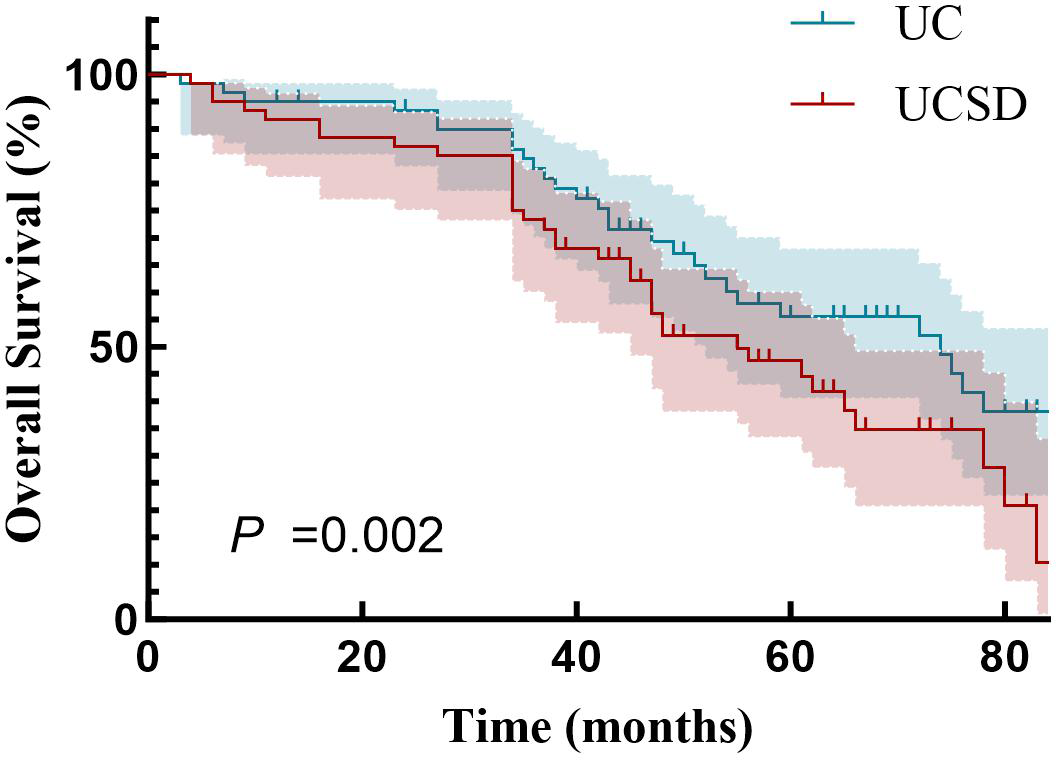
Kaplan-Meier survival curves for overall survival between UC and UCSD patients after propensity score matching. After 1:1 propensity score matching, 60 patients were included in each group. The difference in overall survival between UC and UCSD remained statistically significant (log-rank *P* = 0.002; HR=1.52,95% CI:1.18–1.96).

**Figure 4:**
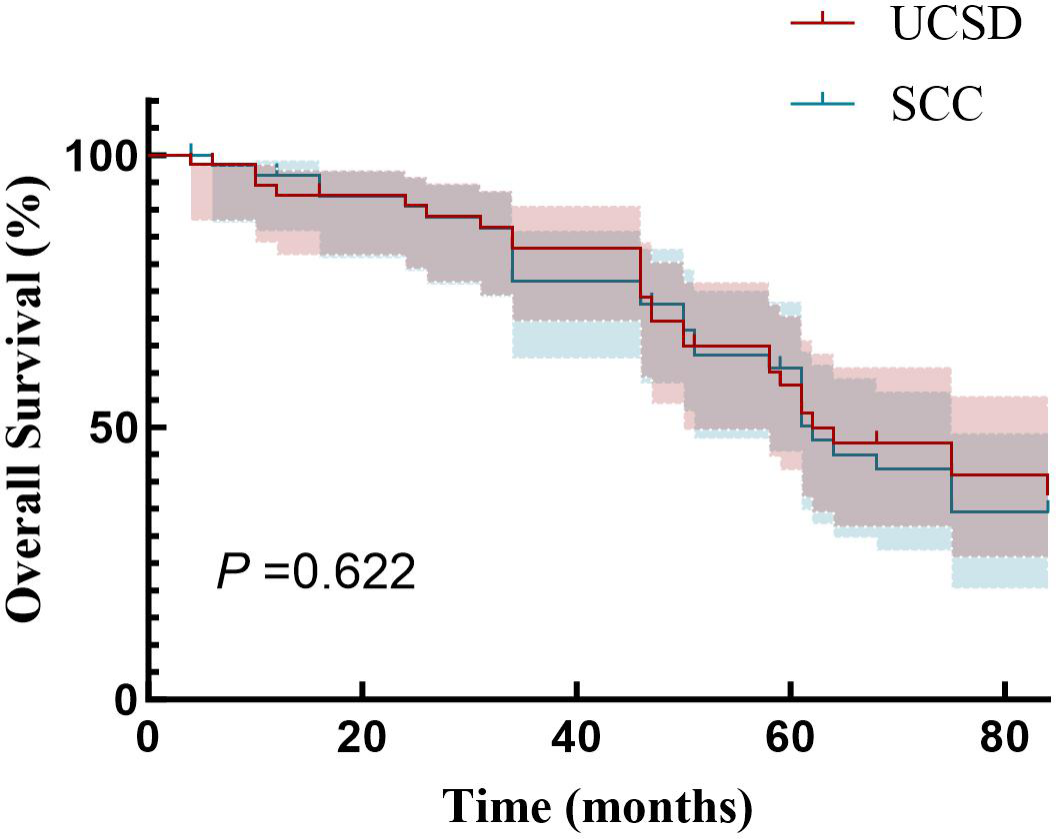
Kaplan-Meier survival curves for overall survival between UCSD and SCC patients after propensity score matching. After 1:1 propensity score matching, 16 patients were included in each group. No significant difference was observed between the UCSD and SCC groups (log-rank *P* = 0.622; HR=1.17, 95% CI:0.79–1.73).

**Figure 5:**
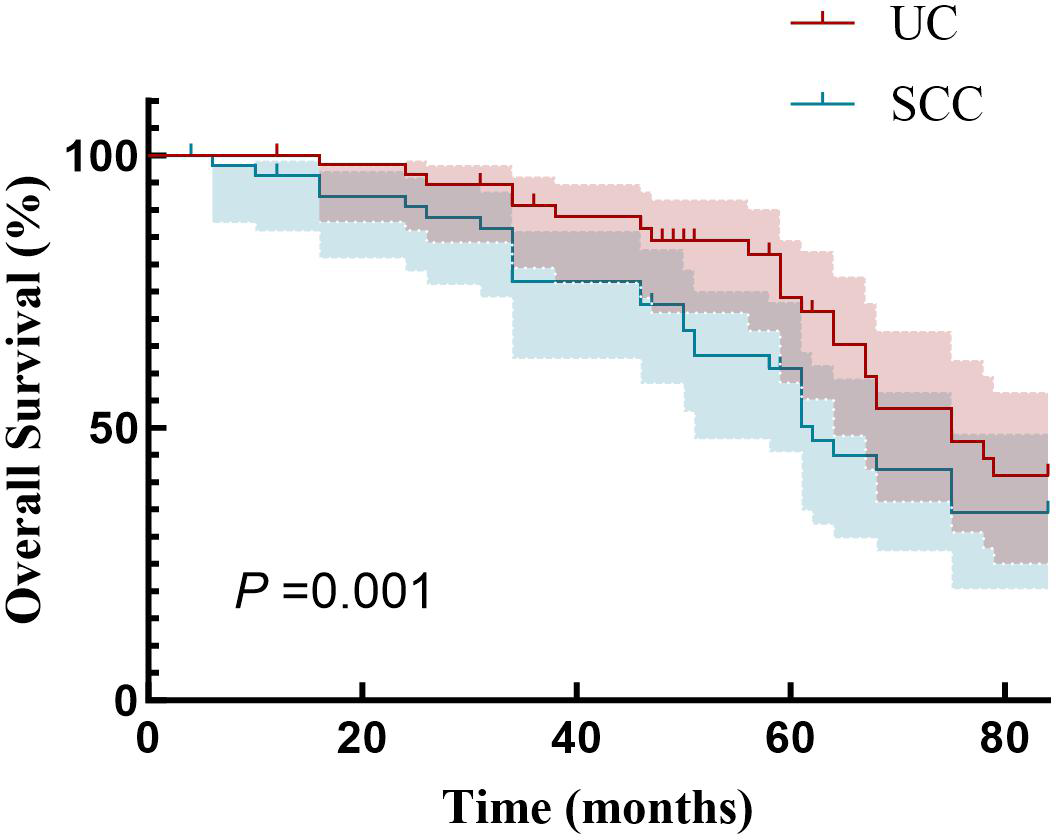
Kaplan-Meier survival curves for overall survival between UC and SCC patients after propensity score matching. After 1:1 propensity score matching, 16 patients were included in each group. The difference in overall survival between UC and SCC remained statistically significant (log-rank *P* = 0.001; HR = 1.78,95% CI:1.25–2.54).

After performing pairwise 1:1 propensity score matching as described in the Methods section, the matched sample sizes were as follows: for the pure UC vs. UCSD comparison, 60 patients in each group (total 120); for pure UC vs. SCC, 16 patients in each group (total 32); and for UCSD vs. SCC, 16 patients in each group (total 32). Pairwise log-rank comparisons demonstrated that overall survival was significantly worse in both the UCSD and SCC groups compared to the pure UC group(UC vs.UCSD: HR = 1.52, 95% CI:1.18–1.96,*P* = 0.002;UC vs.SCC: HR = 1.78,95% CI:1.25–2.54,*P* = 0.001).However, no significant difference was observed between the UCSD and SCC groups(HR = 1.17,95% CI:0.79–1.73,*P* = 0.622) (**Figures 3**–**5**). The corresponding 5-year OS rates were 59.5% for pure UC, 45.8% for UCSD, and 42.6% for SCC. These findings indicate that the adverse survival associated with squamous differentiation persists even after adjusting for key clinicopathological confounders, while the two squamous-containing histologies (UCSD and SCC) have comparable outcomes.

### 3.3 Subgroup analysis of patients with muscle-invasive bladder cancer

To address the substantial between−group difference in the prevalence of muscle invasion, we conducted a subgroup analysis restricted to patients with MIBC (pathological stage≥T2,n=178). Among these patients, the proportion with advanced pathological T stage (T3/T4) was significantly higher in the UCSD (36/49,73.5%) and SCC (11/14,78.6%)groups compared to the pure UC group (56/115, 48.7%;*P* = 0.003).

In multivariable Cox regression analysis limited to the MIBC subgroup and adjusted for T stage (T3/T4vs.T2), lymph node status, age, and adjuvant chemotherapy, both UCSD (HR = 1.48, 95%CI:1.12–1.96,*P* = 0.006) and SCC (HR = 1.71,95% CI: 1.18–2.48, *P* = 0.004) remained independently associated with worse OS compared to pure UC. No significant difference in OS was observed between UCSD and SCC in this subgroup (HR = 1.15,95%CI:0.78–1.70,*P* = 0.482).These findings suggest that the adverse prognostic impact of squamous differentiation is not merely driven by a higher proportion of MIBC but is also evident within the MIBC population after accounting for T stage depth.

### 3.4 Multivariate analysis of prognostic factors

Multivariate analysis was performed using the Cox proportional hazards regression model, incorporating pathological type and other clinicopathological variables. The results of the multivariable Cox regression analysis for the full cohort are summarized in **Table 2**. In the full-cohort model, results demonstrated that increased age (HR = 1.03, 95% CI: 1.02–1.04, *P* < 0.001), muscle invasion (HR = 2.25, 95% CI: 1.75–2.89,*P* < 0.001), positive lymph node status (HR = 2.18, 95% CI: 1.65–2.88,*P* < 0.001), and pathological subtype (both UCSD and SCC) were independent risk factors for overall survival. Specifically, compared to pure UC, UCSD (HR = 1.52, 95% CI: 1.18–1.96,*P* = 0.002) and SCC (HR = 1.78, 95% CI: 1.25–2.54,*P* = 0.001) were independently associated with worse OS. To further assess the difference between UCSD and SCC, a Cox model with UCSD as the reference showed that SCC was not significantly different from UCSD (HR = 1.17, 95% CI: 0.79–1.73,*P* = 0.622). Multivariate analysis conducted in the propensity-score-matched cohort yielded consistent conclusions.

**Table 2:** Multivariate cox regression analysis of factors affecting overall survival in bladder cancer patients (full cohort).

Variable	Hazard ratio (HR)	95% confidence interval (CI)	*P*
Age	1.03	1.02 – 1.04	<0.001
Sex (Male vs. Female)	1.12	0.87 – 1.43	0.380
Pathological type (Ref: Pure UC)			
UCSD	1.52	1.18 – 1.96	0.002
SCC	1.78	1.25 – 2.54	0.001
Muscle Invasion (Yes vs. No)	2.25	1.75 – 2.89	<0.001
Lymph Node Status (Positive vs. Negative)	2.18	1.65 – 2.88	<0.001
Lymphovascular Invasion (Yes vs. No)	1.32	1.01 – 1.73	0.045
Received Adjuvant Chemotherapy (Yes vs. No)	0.85	0.67 – 1.08	0.176

### 3.5 Sensitivity analyses

To assess the robustness of our primary overall survival (OS) findings, we performed two complementary sensitivity analyses.

In the unadjusted cohort, patients with UCSD and SCC had significantly worse 5-year CSS compared to pure UC (48.3% and 44.6%, respectively, vs. 68.2%; log-rank *P* < 0.001). After 1:1 propensity score matching, the differences in CSS among the three groups remained statistically significant for comparisons involving pure UC. Specifically, UCSD vs. pure UC: HR = 1.47, 95% CI: 1.12–1.93,*P* = 0.005; SCC vs. pure UC: HR = 1.69, 95% CI: 1.18–2.42,*P* = 0.004; while UCSD vs. SCC was not significant (HR = 1.15, 95% CI: 0.77–1.72,*P* = 0.481). Multivariable Cox regression for CSS confirmed that pathological subtype was an independent predictor when comparing squamous-containing groups to pure UC.

Given that non-bladder-cancer death could act as a competing event for cancer-specific mortality, we performed a Fine-Gray subdistribution hazard model, treating non-cancer death as a competing risk. After adjusting for the same confounders, the cumulative incidence of bladder cancer-specific death did not differ significantly among the three histologic groups. Compared to pure UC, the subdistribution hazard ratios were 1.44 (95% CI: 1.09–1.90,*P* = 0.011) for UCSD and 1.65 (95% CI: 1.14–2.39, *P* = 0.008) for SCC, while no significant difference was found between UCSD and SCC (SHR = 1.14, 95% CI: 0.75–1.73,*P* = 0.537).

Collectively, these sensitivity analyses confirm that the poorer survival associated with squamous differentiation persists after rigorous adjustment for stage and nodal status, and that squamous histology itself is an independent prognostic factor. The comparable outcomes between UCSD and SCC suggest that the presence of any squamous component, rather than its proportion, drives the adverse prognosis.

### 3.6 External validation results

To assess the generalizability of our primary findings, we performed an external validation using the Surveillance, Epidemiology, and End Results (SEER) database (2004–2020). A total of 4,287 eligible patients who underwent radical cystectomy were identified (pure UC: n=3,562; UCSD: n=498; SCC: n=227). Before matching, significant differences in baseline characteristics were observed, with the UCSD and SCC groups showing more advanced T stage and higher nodal positivity (both *P* < 0.001). After 1:1 propensity score matching (matching for age, sex, T stage, and nodal status), 227 patients were included in each of the three groups, achieving good balance (all SMD <0.1). In the unmatched SEER cohort, Kaplan-Meier analysis demonstrated significantly worse OS for UCSD and SCC compared to pure UC (5-year OS: 45.2% and 41.3% vs. 60.8%, log-rank *P* < 0.001).After 1:1 propensity score matching, pairwise comparisons in the SEER cohort demonstrated that both UCSD and SCC had significantly worse OS compared to pure UC (UCSD vs.UC: HR = 1.33,95% CI: 1.08–1.64,*P* = 0.007; SCC vs.UC: HR = 1.51, 95% CI:1.15–1.98, *P* = 0.003), whereas no significant difference was observed between UCSD and SCC (HR = 1.14, 95% CI: 0.86–1.51, *P* = 0.369). Multivariable Cox regression in the matched SEER cohort confirmed that pathological subtype was an independent predictor of OS for comparisons with pure UC. These external validation results are consistent with our primary single-center findings, supporting the robustness of the conclusion that squamous differentiation itself is an independent prognostic factor after adjusting for key clinicopathological confounders, with comparable outcomes between UCSD and SCC.

## 4 Discussion

The clinical management of bladder cancer is highly dependent on precise risk stratification (13). This retrospective study confirmed that bladder cancer with squamous differentiation presents with more aggressive features at diagnosis, including advanced pathological stage,higher rates of muscle invasion, and greater risk of lymph node metastasis – findings consistent with most previous reports (14–17). However, the key question has been whether this aggressive behavior is merely a reflection of later presentation or whether squamous differentiation itself independently worsens prognosis.

After rigorously balancing key confounders(age, sex, surgical approach, muscle invasion status, lymph node status, and tumor diameter)through 1:1 propensity score matching, we observed that both UCSD and pure SCC remained associated with significantly worse overall survival compared to pure UC(UC vs.UCSD: HR = 1.52, 95% CI:1.18–1.96, *P* = 0.002;UC vs.SCC: HR = 1.78, 95% CI: 1.25–2.54,*P* = 0.001).In the matched cohort, the 5−year OS rates were 59.5% for pure UC, 45.8% for UCSD, and 42.6% for SCC.In contrast, no significant survival difference was found between UCSD and SCC (HR = 1.17, 95% CI: 0.79–1.73,*P* = 0.622). Multivariable Cox regression further confirmed that pathological subtype (UCSD or SCC)was an independent predictor of worse OS, even after adjusting for traditional prognostic factors. These findings were replicated in an external validation using the SEER database, where pairwise comparisons after PSM still showed significantly worse OS for UCSD and SCC versus pure UC, with comparable outcomes between the two squamous-containing groups.

Collectively, our results provide strong evidence that squamous differentiation itself is an independent adverse prognostic factor in bladder cancer patients undergoing radical cystectomy.The persistent survival disadvantage after rigorous adjustment argues against the notion that poor outcomes are merely a consequence of later stage at presentation. Instead, the presence of squamous elements appears to confer intrinsically more aggressive tumor biology that is not fully captured by TNM staging or nodal status.

Furthermore, to directly address the concern that the poor prognosis associated with squamous differentiation might be largely attributable to a higher prevalence of muscle-invasive disease, we performed a subgroup analysis confined to MIBC patients. Even within this cohort, UCSD and SCC exhibited significantly more advanced T stage (T3/T4) compared to pure UC. After adjusting for T stage depth and nodal status, both UCSD and SCC remained independent predictors of worse overall survival, whereas no survival difference was observed between the two squamous-containing groups. These results reinforce the conclusion that squamous differentiation confers an intrinsically more aggressive tumor biology beyond its association with muscle invasion.

Nakagawa et al. analyzed 99 patients with pT1N0M0 urothelial carcinoma treated conservatively with transurethral resection and found that UCSD or glandular differentiation was independently associated with significantly worse overall survival (OS) and cancer−specific survival (CSS) compared to pure UC; notably, these patients experienced markedly poorer outcomes after stage progression, leading the authors to recommend early radical cystectomy for this population (9). This aligns with our observation that squamous differentiation confers adverse prognosis even in lower−stage disease, suggesting that its negative impact is not solely driven by advanced stage.

In the muscle−invasive setting, a Korean multi−institutional study of 432 patients undergoing radical cystectomy for MIBC demonstrated that UCSD had a 5−year OS of only 41.1% compared to 69.7% for pure UC, with squamous differentiation identified as an independent predictor of OS in multivariate analysis(HR = 4.22) (14).However, other studies have yielded conflicting results. Mitra et al., after controlling for pathological stage and nodal status using propensity score matching in a cohort of 934 patients, reported that squamous differentiation was not associated with an increased risk of disease−specific mortality (HR = 1.35,95% CI: 0.90–2.04) (17). Similarly, in a multi−institutional study of 1082 patients undergoing upfront RC, Claps et al. found that among various variant histologies, squamous differentiation was not significantly associated with worse disease−specific survival compared to pure UC after adjusting for pathological stage and nodal status, in contrast to small−cell or sarcomatoid variants which retained independent prognostic significance (8).

These discrepancies may be attributable to several factors, including differences in the proportion of patients receiving neoadjuvant chemotherapy, variability in pathological staging rigor, and heterogeneity in the definition of squamous differentiation. In the present study, employing both propensity score matching and external validation in the SEER cohort, we observed that after rigorous adjustment for key clinicopathological confounders, UCSD and SCC remained significantly associated with worse OS compared to pure UC, with HRs of 1.52 and 1.78, respectively. These findings provide robust evidence that the adverse prognostic impact of squamous differentiation extends beyond what can be explained by stage and nodal status alone.

Accumulating evidence suggests that bladder cancers harboring squamous differentiation exhibit distinct molecular characteristics that may influence their response to conventional cisplatin−based chemotherapy. The basal/squamous molecular subtype, which is closely associated with squamous histology, is characterized by elevated expression of programmed death−ligand 1(PD−L1), enrichment of immune cell infiltration, and activation of epithelial−mesenchymal transition pathways (18).A post−hoc analysis of the GETUG/AFU VESPER trial, which included 300 patients with muscle−invasive bladder cancer treated with neoadjuvant cisplatin−based chemotherapy, demonstrated that tumors of the basal/squamous (pure or mixed) molecular subtype had significantly worse progression−free survival compared to other subtypes, with a hazard ratio of 2.0 (95% CI:1.36–3.0,*P* < 0.001).Furthermore, mixed tumors—many of which contained a basal/squamous component—were associated with decreased metabolic activity that could account for chemoresistance, and basal/squamous non−responders largely maintained their subtype at the time of cystectomy.

Consistent with these molecular observations, the Korean study reported that UCSD exhibits a poor response to cisplatin−based chemotherapy compared to pure UC (14). The VESPER trial also found that the proportion of patients achieving pathological downstaging did not differ significantly between variant histology and pure UC overall; however, patients with tumors containing>50% squamous cell subtype were associated with decreased progression−free survival at three years compared to those with pure UC.These findings suggest that while squamous differentiation itself does not preclude the use of neoadjuvant chemotherapy—and current guidelines recommend a similar approach as for pure UC—the presence of a substantial squamous component may portend a less favorable response and warrants further investigation into alternative perioperative strategies.

Emerging data on immune checkpoint inhibitors provide additional therapeutic avenues. Basal/squamous tumors are characterized by elevated PD−L1 expression and an inflamed tumor microenvironment, and in squamous bladder cancer specifically, hot tumor−immune phenotypes with strong PD−L1 expression have been identified as a promising subgroup for immune checkpoint inhibitor therapy (18).These observations support the evaluation of immunotherapeutic approaches, either as monotherapy or in combination with chemotherapy, for patients with UCSD or SCC in future clinical trials.

While the present study was not designed to directly compare treatment strategies, the following clinically actionable messages can be drawn from our findings.First, risk stratification following radical cystectomy should not rely solely on TNM stage and nodal status; pathological identification of squamous differentiation—whether as UCSD or pure SCC—should be incorporated into prognostic models to identify patients at higher risk of death. Second, given the potential chemoresistance associated with basal/squamous molecular subtypes, as observed in the VESPER trial, conventional cisplatin−based regimens may be less effective in tumors with a substantial squamous component. Alternative perioperative strategies warrant consideration. Notably, bladder−preserving non−surgical management in non−organ−confined non−urothelial bladder cancer is associated with significantly higher cancer−specific mortality (70.4% vs.60.6% at five years) compared to radical cystectomy, and this difference was driven primarily by SCC patients (HR = 2.80), reinforcing the central role of radical cystectomy in operable SCC patients (7).For early−stage disease,Deuker et al. demonstrated that radical cystectomy for T1N0M0 SCC provided a cancer−specific survival benefit, with 60−month CSS of 86% in the RC group versus 66% in non−RC patients (7).

Third, the consistently higher stage at diagnosis among patients with squamous differentiation underscores the need for early detection strategies, particularly in at−risk populations including women and patients with chronic urinary tract infections or schistosomiasis. Fourth, from a pathological reporting perspective, any squamous component—even if focal — should be clearly documented, as it carries independent prognostic weight. Although the optimal treatment for UCSD and SCC remains to be defined in prospective trials, our findings support the inclusion of these patients in studies evaluating novel perioperative therapies, including immune checkpoint inhibitors and antibody−drug conjugates, given the molecular rationale derived from basal/squamous subtyping.

Recent advances have linked squamous differentiation to specific molecular subtypes of bladder cancer. Squamous histology is frequently associated with the basal molecular subtype, which is characterized by elevated expression of programmed death-ligand 1 (PD-L1), enrichment of immune cell infiltration, and activation of epithelial-mesenchymal transition pathways (18). These features may contribute to chemotherapy resistance and a more aggressive clinical course. In our study, the independent prognostic impact of squamous differentiation after PSM suggests that such molecular drivers – rather than simply advanced stage – are at play. Moreover, the lack of survival difference between UCSD and pure SCC implies that the presence of any squamous component, regardless of its proportion, is the key determinant of poor prognosis. This aligns with the concept that even focal squamous differentiation can drive a basal-like phenotype.

Our findings contradict several earlier reports that concluded squamous differentiation loses prognostic significance after adjusting for stage (10, 17). The discrepancy likely stems from methodological differences. Many prior studies had limited sample sizes, especially for pure SCC, and did not employ rigorous propensity score matching that simultaneously accounts for multiple confounders. Additionally, some studies included non-muscle-invasive tumors or heterogeneous treatments (e.g., transurethral resection alone), whereas our cohort was uniformly treated with radical cystectomy. By using pairwise PSM and external validation, we provide more robust evidence that the adverse prognosis associated with squamous differentiation is independent of stage and nodal status.

Conversely, our results support studies that identified squamous differentiation as an independent predictor of recurrence or cancer-specific mortality (9, 14). For instance, Nakagawa et al. found that squamous or glandular differentiation predicted poor prognosis in pT1 urothelial carcinoma (9). The consistency between our findings and those reports strengthens the conclusion that squamous histology should be considered a high-risk feature.

It is worth noting that other variant histologies, such as sarcomatoid or micropapillary differentiation, have also been reported to confer poor prognosis independent of stage (19, 20). The molecular heterogeneity across these variants suggests that different biological drivers may be operative. Nonetheless, our findings specifically address squamous differentiation – one of the most common divergent patterns–and provide a clear signal that it should be managed as an adverse feature.

The major strengths of this study include the relatively large sample size for UCSD, rigorous pairwise propensity score matching to minimize confounding, centralized pathological review with high inter-observer agreement (κ=0.87), and external validation using the SEER database. The consistent results across internal and external cohorts enhance the generalizability of our conclusions.

Several limitations must be acknowledged.First, as a retrospective study, selection bias and residual confounding cannot be completely eliminated, although PSM addressed measured confounders.Second, we lacked detailed information on neoadjuvant chemotherapy (NAC). Differential administration of NAC across histological subtypes could affect survival. However, the persistent independent effect of squamous differentiation after PSM suggests that histology-driven biological differences are not fully explained by treatment variation. Future prospective studies should collect standardized NAC data.Third, the sample size of pure SCC (n=16) in our institutional cohort was relatively small, which may affect the precision of hazard ratio estimates. Nevertheless, the external SEER validation (n=227 for SCC) confirmed the same pattern, mitigating this concern.Fourth, the percentage of squamous differentiation within UCSD tumors was not quantitatively recorded. Although we found no difference between UCSD and pure SCC, it remains possible that a threshold effect exists (e.g., only >10% squamous component drives poor prognosis). Future studies should adopt standardized reporting of the proportion of squamous elements. Fifth, we did not perform molecular subtyping or PD-L1 immunohistochemistry on our cohort. The proposed biological mechanisms (basal subtype, immune evasion) are therefore speculative and should be directly tested in future translational studies.

Our findings open several avenues for further research. First, prospective multicenter cohorts with systematic collection of NAC data, standardized quantification of squamous differentiation, and integrated molecular profiling are needed to validate and extend our results. Second, randomized trials should explore whether patients with squamous-containing tumors derive differential benefit from adjuvant immunotherapy versus chemotherapy. Third, the development of predictive biomarkers could help refine treatment selection for this high-risk subgroup.

## 5 Conclusion

In summary, both UCSD and pure SCC are independently associated with poorer prognosis after radical cystectomy, even after rigorous adjustment for stage, nodal status, and other confounders using propensity score matching. The adverse survival impact persists in external validation using the SEER database, confirming that squamous differentiation is an independent risk factor. However, no significant difference in survival exists between UCSD and pure SCC, suggesting that the presence of any squamous component is the key determinant. These findings support incorporating squamous differentiation into risk stratification models and clinical trial eligibility criteria, and they underscore the need for further research into molecular mechanisms and targeted therapies for squamous histologies in bladder cancer. Nevertheless, given the limited sample size of pure SCC in our institutional cohort, further studies with larger SCC cohorts are warranted to confirm these results. The diversity and heterogeneity of bladder cancer determine the complexity of its clinical management, particularly for subtypes with variant histological features. Future studies should further investigate the biological and genomic characteristics of different pathological subtypes and their responses to various adjuvant treatments, aiming to improve patient survival and quality of life through strategies such as early diagnosis and precision therapy.

## Data Availability

The raw data supporting the conclusions of this article will be made available by the authors, without undue reservation.
